# The global pediatric nephrology workforce: a survey of the International Pediatric Nephrology Association

**DOI:** 10.1186/s12882-016-0299-2

**Published:** 2016-07-15

**Authors:** Dorey Glenn, Sophie Ocegueda, Meaghan Nazareth, Yi Zhong, Adam Weinstein, William Primack, Pierre Cochat, Maria Ferris

**Affiliations:** UNC Kidney Center, University of North Carolina at Chapel Hill, 7024 Burnett-Womack, Chapel Hill, NC 27599-7155 USA; Division of Nephrology, Children’s Hospital at Dartmouth, 1 Medical Center Drive, Lebanon, NH 03766 USA; Service de Néphrologie Rhumatologie Dermatologie, Hôpital Femme Mère Enfant & Université Claude-Bernard, Lyon 1, 69677 Bron Cedex, France

**Keywords:** Pediatric, Nephrology, Workforce, Training, Global health, Survey

## Abstract

**Background:**

The global pediatric nephrology workforce is poorly characterized. The objectives of our study were to assess pediatric nephrologists’ perceptions of the adequacy of the pediatric nephrology workforce, and understand regional challenges to fellow recruitment and job acquisition. Perceptions regarding optimal length of training and research requirements were also queried.

**Methods:**

A 17-question web-based survey comprised of 14 close-ended and 3 open-ended questions was e-mailed to members of the International Pediatric Nephrology Association. Quantitative and qualitative analyses were performed.

**Results:**

We received 341 responses from members of the International Pediatric Nephrology Association from 71 countries. There was a high degree of overall perceived workforce inadequacy with 67 % of all respondents reporting some degree of shortage. Perceived workforce shortage ranged from 20 % in Australia/New Zealand to 100 % in Africa. Respondents from Africa (25 %) and North America (22.4 %) reported the greatest difficulty recruiting fellows. Respondents from Australia/New Zealand (53.3 %) and Latin America (31.3 %) reported the greatest perceived difficulty finding jobs as pediatric nephrologists after training. Low trainee interest, low salary, lack of government or institutional support, and few available jobs in pediatric nephrology were the most frequently reported obstacles to fellow recruitment and job availability.

**Conclusions:**

Globally, there is a high level of perceived inadequacy in the pediatric nephrology workforce. Regional variability exists in perceived workforce adequacy, ease of recruitment, and job acquisition. Interventions to improve recruitment targeted to specific regional barriers are suggested.

**Electronic supplementary material:**

The online version of this article (doi:10.1186/s12882-016-0299-2) contains supplementary material, which is available to authorized users.

## Background

Workforce and training data are important for resource allocation and strategic planning. There is a paucity of published literature describing the adequacy of the pediatric nephrology (PN) workforce on a global scale [1–4].

Kidney disease is increasingly recognized as an important contributor to the global burden of disease [5–9]. In 2012 chronic kidney disease was ranked 18^th^ in a report on global burden of death [5]. The demand for pediatric nephrologists (PNs) is likely to further expand as the pattern of non-communicable disease in developing countries continues to change and economic development in those regions is able to support a greater range of health care interventions [10, 11]. The availability of maintenance dialysis, for example, has increased substantially from 1990 to 2010 [6]. The role of pediatric nephrologists, however, extends far beyond the care of patients with acute and chronic kidney disease. PNs support intensive care units, treat complications from non-renal diseases, and fill important educational and administrative roles [1, 12, 13].

Concern regarding the long term PN workforce within the United States is based on a high number of potential retirees in the field and difficulty recruiting trainees [1]. For example, in 2015 only 37 of 58 available fellowship positions were filled [14]. However, to our knowledge, perceived adequacy of the PN workforce have not been systematically described in a context external to the United States.

Some attention has been directed towards the global nephrology workforce shortage by adult nephrologists [9, 15–17]. Efforts to develop training curricula and expand training opportunities to physicians from resource-limited settings have been made on the part of the International Society of Nephrology and the International Pediatric Nephrology Association (IPNA) for example [15, 16]. The objectives of our study are to describe the perceived adequacy of the PN workforce and understand regional challenges to fellow recruitment and job acquisition.

## Methods

### Survey development

We sought to develop and administer a survey instrument assessing the global PN workforce. Prior to survey development, a MEDLINE search to identify prior evaluations of the global PN workforce was performed, and none were identified. We developed an English language, web-based survey assessing 3 domains: trainee recruitment, job availability, and workforce adequacy. Survey questions were developed with the input of research team members. A draft survey instrument was pre-tested by 5 pediatric nephrologists to assess content validity, interpretability, and length. The draft instrument was updated based on pretesting feedback. The final survey instrument was comprised of 14 close-ended and 3 open-ended questions. Question response types included dichotomous, multiple choice, Likert scale, and free text responses. The survey was developed and deployed via the Qualtrics platform at the University of North Carolina at Chapel Hill (see Additional files 1, 2 and 3) [18]. This study was deemed exempt by the Human Research Ethics Committee of the University of North Carolina Chapel Hill.

### Survey administration

Survey invitations were sent through e-mail to non-North American IPNA members with a valid e-mail address in the online IPNA directory in the fall of 2015. One reminder e-mail was sent 2 weeks later. Survey invitations were sent to North American IPNA members in December of 2015. Implied consent was obtained prior to survey participation. Survey participants received no individual remuneration; however, a donation to IPNA was made on their behalf.

### Statistical analysis

Quantitative data was analyzed using STATA version 13 (College Station, TX) and Microsoft Excel 2013 (Redmond, WA). Survey respondent data were reported as counts and proportions for categorical variables, and medians and interquartile ranges for continuous variables. T-tests were performed comparing ratios of workforce shortage, difficulty recruiting fellows, and difficulty finding a PN job for each region compared to all other regions. T-tests were performed with the allowance for unequal variances. Qualitative analyses were performed using ATLAS.ti version 7 (Berlin, Germany). Thematic analysis was independently performed by two research team members (SO and MN) to code open-ended questions into 11 themes. Discrepancies between coders were rectified by a third party (DG). Investigator triangulation was used to ensure that the identified themes reflected the range and depth of responses. All survey responses were collected and analyzed anonymously.

## Results

### Survey respondents

We received 341 responses from 2304 valid e-mail invitations, yielding an overall response rate of 15 %. Responses were received from 71 countries. Regional response rates ranged from 11.2 % in Asia to 31.3 % in Australia and New Zealand.

**Fig. 1:**
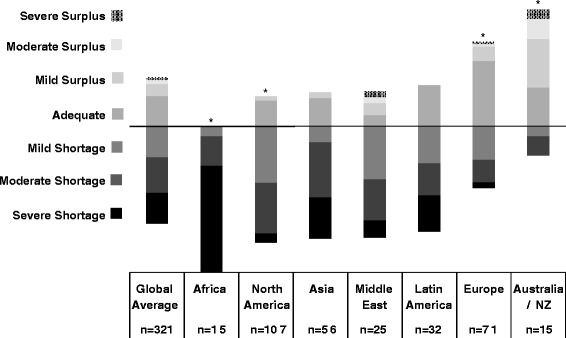
Perceived adequacy of the PN workforce by region and * indicates a significantly different (*p* < 0.05) ratio of respondents reporting workforce shortage compared to the average of other regions

**Fig. 2:**
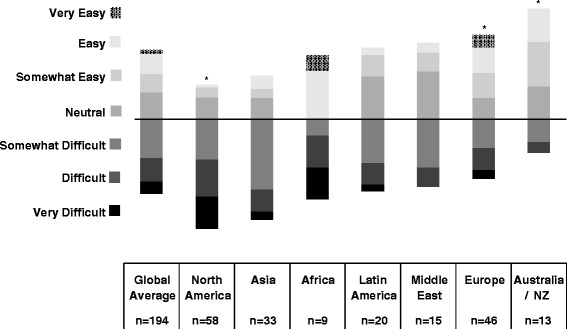
Perceived difficulty recruiting trainees by region and * indicates a significantly different (*p* < 0.05) ratio of respondents reporting recruitment difficulty compared to the average of other regions

**Fig. 3:**
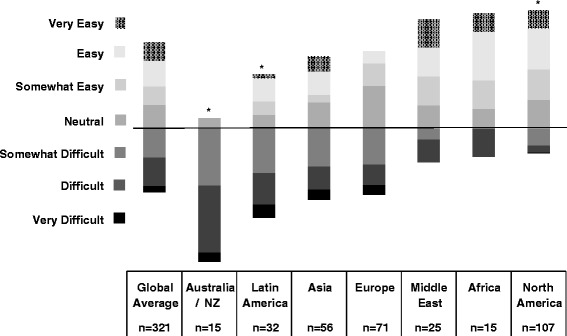
Perceived difficulty finding a PN job after training by region and * indicates a significantly different (*p* < 0.05) ratio of respondents reporting a perceived difficulty with job acquisition after training compared to the average of other regions

**Fig. 4:**
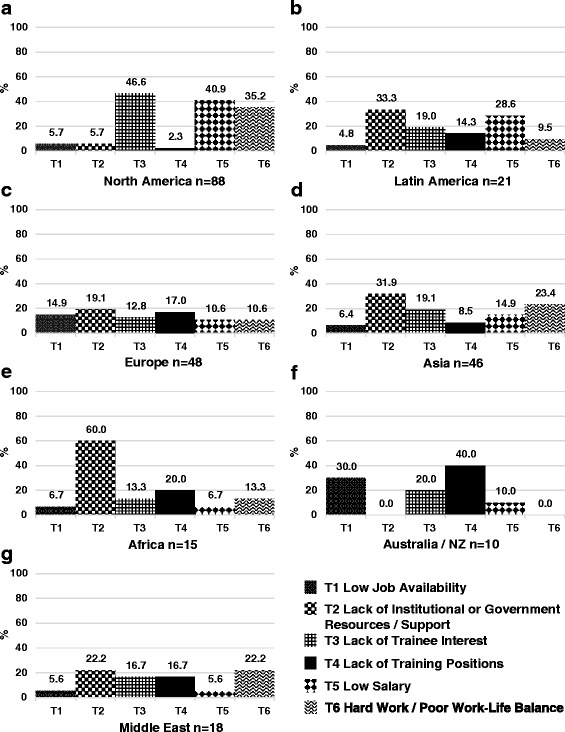
Results of qualitative analysis of responses to challenges to PN trainee recruitment in North America (**a**), Latin America (**b**), Europe (**c**), Asia (**d**), Africa (**e**), Australia and New Zealand (**f**), and the Middle East (**g**)

**Fig. 5:**
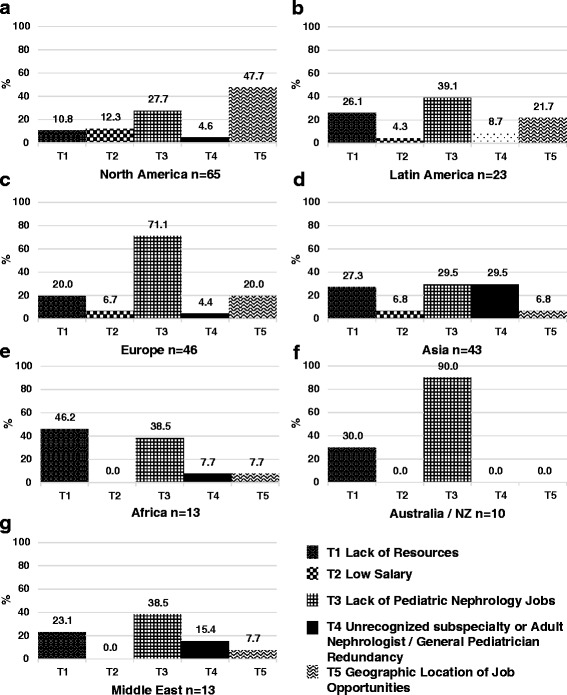
Results of qualitative analysis of responses to challenges to job acquisition after PN training in North America (**a**), Latin America (**b**), Europe (**c**), Asia (**d**), Africa (**e**), Australia and New Zealand (**f**), and the Middle East (**g**)

Tables 1 and 2 display region, practice type, involvement in trainee education, and percent time allocated to clinical practice and research for the survey respondents. Of the 341 respondents, 167 (48 %) were affiliated with an academic or university based practice and 206 (60 %) reported involvement in trainee education. The median time percentage allocated to clinical nephrology and research was 66.7 % (IQR 50.0-75.0) and 25.0 % (IQR 0–25.0), respectively. Figures 1, 2, and 3 represent the perceived adequacy of the PN workforce, difficulty recruiting trainees, and difficulty finding a PN job after training by region. Figures 4 and 5 display results of qualitative analysis of 456 open-ended responses querying challenges to PN trainee recruitment and job acquisition.

**Table 1:** Survey respondent and non-respondent characteristics^a^

	Survey respondents	Survey non-responders
Region		
North America	107 (31.4)	566 (28.8)
Latin America	40 (11.7)	225 (11.5)
Europe	75 (22.0)	450 (22.9)
Asia	61 (17.9)	480 (24.5)
Africa	17 (5.0)	66 (3.4)
Australia/New Zealand	15 (4.4)	33 (1.7)
Middle East	26 (7.6)	143 (7.3)
Total	341	1963
Practice Type		
Academia or University Based	167 (49.0)	
Government Affiliated	81 (23.8)	
Multiple Practice Types	65 (19.1)	
Private Practice	17 (5.0)	
Other	9 (2.6)	
Military Affiliated	2 (0.6)	
Involved in Trainee Education		
Yes	206 (60.4)	
No	135 (39.6)	

^a^Values presented as n (%)

**Table 2:** Percent time allocated to clinical pediatric nephrology, research, and other activities by region^a^

	*n*	Clinical Pediatric Nephrology	Research	Other
Region				
North America	107	50.0 (50.0–75.0)	25.0 (0–25.0)	20.0 (0–25.0)
Latin America	39	66.7 (50.0–75.0)	20.0 (0–28.6)	14.3 (0–25.0)
Europe	75	75.0 (50.0–75.0)	25.0 (0–25.0)	20.0 (0–25.0)
Asia	59	50.0 (40.0–75.0)	20.0 (0–25.0)	25.0 (0–25.0)
Africa	17	50.0 (50.0–75.0)	25.0 (25.0–37.5)	0 (0–25.0)
Australia/New Zealand	15	75.0 (50.0–100.0)	0 (0–25.0)	0 (0–25.0)
Middle East	26	75.0 (50.0–75.0)	25.0 (16.7–25.0)	0 (0–25.0)
Total	338^b^	66.7 (50.0–75.0)	25.0 (0–25.0)	14.3 (0–25.0)

^a^Values presented as median and interquartile range (IQR)

^b^Three respondents who did not report their time breakdown (1 from Latin America and 2 from Asia) were excluded from the analysis

### North America

A total of 107 responses (response rate of 15.9 %) were received from North American PNs (100 from the United States, 7 from Canada). Mexico was included in the Latin American region. Seventy nine percent of North American respondents reported a mild to severe shortage in the PN workforce (Fig. 1). Of those expressing some degree of shortage, 52 % reported a moderate to severe shortage. North American respondents reported the greatest difficulty recruiting trainees with 76 % reporting this activity to be somewhat to very difficult (Fig. 2). Lack of interest (47 %), low salary (41 %), and hard work/poor work-life balance (35 %) were the most frequently reported challenges to trainee recruitment. See Fig. 4. Sixty three percent of North American respondents reported it to be somewhat easy to very easy to find a PN job after training, but 48 % noted that geographic distribution of jobs was a challenge to job acquisition (Figs. 3 and 5).

### Africa

Seventeen responses (response rate 20.7 %) were received from nine African countries. All respondents reported some degree of workforce shortage and 71 % reported a severe shortage. Of the 8 respondents involved in physician training, there was variation in perceived ease regarding trainee recruitment. Lack of institutional or government support and resources was the most frequently sighted reason for difficulty recruiting trainees in respondents from Africa (60 %). The majority (64 %) also reported it to be somewhat to very easy to find a job after training. Barriers to job acquisition after training included lack of resources (46 %) and lack of PN positions (39 %).

### Australia/New Zealand

Fifteen responses (response rate 31.3 %) were received from Australia and New Zealand. In contrast to North America, 53 % of respondents reported a mild to severe surplus of PNs, and 27 % thought the workforce was adequate. Relative to all other regions, respondents from Australia and New Zealand reported the greatest ease recruiting fellows, 54 % reporting recruitment to be somewhat to very easy. A lack of training positions (40 %) and low job availability (30 %) were cited as challenges to fellow recruitment. Consistent with the reported relative surplus of PNs, 93 % of respondents reported it to be somewhat to very difficult to find a job after training with 90 % citing a lack of available PN positions as the major obstacle to job acquisition.

### Europe

Seventy five responses (response rate 14.4 %) were received from 26 European countries. Responses from Russia were included as part of the European region. Fifty eight percent of respondents from Europe felt the PN workforce was adequate or in surplus, which is substantially higher than other regions (with the exception of Australia/NZ). Forty two percent reported a mild to severe shortage. Of the 67 % of European respondents involved in physician training, 41 % reported difficulty, 43 % reported ease, and 15 % were neutral regarding their ability to recruit trainees. More respondents reported difficulty (46 %) than ease (25 %) with job acquisition following training, and 30 % were neutral on this issue. A perceived lack of jobs (71 %), lack of resources (20 %), and the geographic location of jobs (20 %) were the most frequently reported challenges to job acquisition.

Of the 19 respondents from Eastern Europe, 63 % reported a perceived workforce shortage. This was significantly different (*p* = 0.038) from respondents in Western Europe (34.6 %, *n* = 52). There were no significant differences in the percent of respondents from these two sub-regions reporting difficulty recruiting trainees (*p* = 0.978) or difficulty finding a PN job after training (*p* = 0.127).

### Latin America

Forty responses (response rate 15.1 %) were received from 13 countries in Latin America. For the purposes of our study, we included Jamaica and Mexico as part of Latin America. Twenty eight percent of respondents from Latin America felt the PN workforce was adequate in their country, while 72 % reported a mild to severe shortage. Of the 63 % of respondents involved in physician training, 50 % reported difficulty, 20 % reported ease, and 30 % were neutral regarding their ability to recruit trainees. Lack of institutional or government support and resources (33 %) and low salary (29 %) were the most frequently reported challenges to trainee recruitment. Two thirds of respondents perceived job acquisition following training to be somewhat to very difficult. The perceived difficulty obtaining a PN job after training in Latin America was significantly higher than the average of other regions. Lack of available PN positions (39 %), lack of resources (26 %), and geographic location of jobs (22 %) were the most frequently reported challenges to job acquisition.

### Asia

Sixty one responses (response rate 11.2 %) from 12 Asian countries were received. Countries in the Middle East were excluded from the Asian region and analyzed separately. Seventy seven percent of respondents from Asia reported a mild to severe shortage in the PN workforce. Of the 57 % of Asian respondents involved in physician training, 70 % reported difficulty, 15 % reported ease, and 15 % were neutral regarding their ability to recruit trainees. Lack of institutional or government support and resources (32 %) and hard work/poor work-life balance (23 %) were the most frequently reported challenges to trainee recruitment. Fifty percent of respondents perceived job acquisition following training to be somewhat to very difficult, and 32 % reported it to be somewhat to very easy. Lack of available PN positions (30 %), lack of resources (27 %), and poor subspecialty recognition/adult nephrologist redundancy (30 %) were the most frequently reported challenges to job acquisition after training.

### Middle East

Twenty six responses from 8 countries were received (response rate 15.3 %). Turkey was included in the Middle East region rather than Europe or Asia. Seventy six percent of respondents from the Middle East reported a mild to severe shortage in the PN workforce. Of the 58 % respondents from the Middle East involved in physician training, 47 % reported difficulty, 20 % reported ease, and 33 % were neutral regarding their ability to recruit trainees. Lack of institutional or government support and resources (22 %) and hard work/poor work-life balance (22 %) were the most frequently reported challenges to trainee recruitment. Sixty percent of respondents from the Middle East reported it to be somewhat easy to very easy to find a PN job after training. Lack of available PN positions (39 %) and lack of resources (23 %) were the most frequently reported challenges to job acquisition after training.

### Training duration and research requirements

**Fig. 6:**
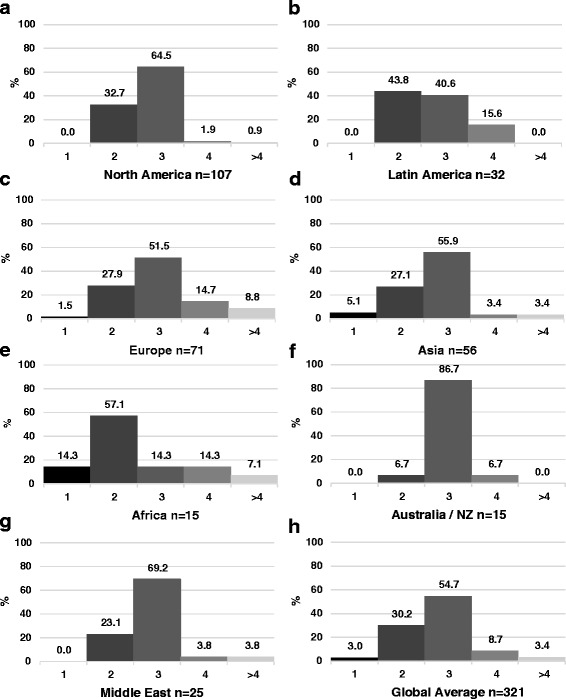
Optimal years of pediatric nephrology training in North America (**a**), Latin America (**b**), Europe (**c**), Asia (**d**), Africa (**e**), Australia and New Zealand (**f**),  Middle East (**g**), and the global average (**h**)

**Fig. 7:**
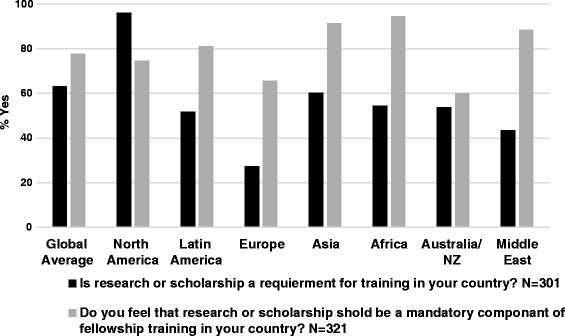
Actual and desired research requirement in PN training by region

Three years of PN training was perceived as optimal by the majority of respondents in North America, Europe, Asia, Australia/New Zealand, and the Middle East (Fig. 6). The majority of respondents from Latin America and Africa reported that 2 years of PN training is optimal. The percentage of respondents reporting mandatory research requirements in their country varied from 27 % in Europe to 96 % in North America (Fig. 7). In all regions, apart from North America, the percentage of respondents reporting that research or scholarship should be a mandatory part of PN training was greater than the percentage reporting a current research requirement.

## Discussion

To our knowledge, this survey represents the first global assessment of the PN workforce. PNs in most regions of the world perceive that the PN workforce is inadequate, with the exception of Australia/NZ and certain areas of Europe, where the workforce is felt to be adequate or even in surplus. Fifty three percent of respondents from Australia and New Zealand reported a mild to severe surplus of PNs, with an additional 27 % reporting the workforce as adequate. Among all regions, respondents from Australia/NZ also reported the greatest difficulty with job acquisition and greatest ease with fellow recruitment. These data suggest an oversupply and/or highly interested pool of potential PN trainees relative to training slots, and a potentially over-saturated or mal-distributed PN job market.

Similarly, though to a lesser extent than Australia/NZ, the majority (58 %) of European respondents reported workforce adequacy or surplus. Regional variability regarding workforce adequacy has been noted in the adult nephrology community in Europe [15]. Sharif et al. speculated that this variability might be explained by differences in healthcare delivery models, matching of workforce supply to service demands, and the use of physician extenders [15]. We were unable to verify the applicability of these factors to the PN workforce from our data. Factors affecting the supply of adult nephrologists may not translate to the PN workforce due to, for example, contrasting distributions in the public-private sectors and differing training requirements. Our findings from Europe and Australia/NZ stand in sharp contrast, however, to North America, Latin America, Asia, the Middle East, and Africa.

Neither the high degree of perceived workforce inadequacy in North America, nor the challenges to trainee recruitment identified by North American respondents, are surprising [1, 12, 19]. Lack of interest, hard work/poor work-life balance, and poor salary were obstacles to fellow recruitment reported by more than a third of North American respondents. These themes echo the results of a 2008 survey of U.S pediatric nephrology fellows that reflected lack of interest, financial constraints, and perceived PN workload as factors dissuading potential trainees from PN [20]. Similar themes have been identified as obstacles in the adult nephrology workforce [15, 17]. In our data, these themes were more frequently reported in North America than in any other region, suggesting that interventions in North America need to be directed toward addressing these obstacles.

Contrasting perceptions between respondents from North America and Europe are particularly noteworthy. Seventy nine percent of North American respondents reported a perceived workforce inadequacy, compared to 42 % of European respondents. Similarly, difficulty recruiting trainees was reported by 76 % of North American respondents, compared to 41 % of European respondents. While our data cannot be used to substantiate objective differences in the PN workforce between these two regions, we hypothesize that perceived differences might be related to variation in the use of physician extenders, the referral patterns of primary care providers, and the relative ease of recruiting and utilizing trainees. Additional research is needed to replicate and further explore these findings.

### Suggested interventions to improve the pediatric nephrology workforce

#### Length of training and mandatory research requirements

Length of training and mandatory research requirements are two factors that might be modified to increase the appeal of PN to potential trainees. The Latin American and African regions were the only regions, however, to prefer 2 years of training over 3. The data also suggest a greater interest in increasing, rather than decreasing, research/scholarship requirements in all regions other than North America.

In our data, 75 % of North American respondents felt that research or scholarly activity should be mandatory; a notable finding given that research is currently a required component of PN fellowship training in the United States. Some North American respondents commented that mandatory research requirements might be a barrier to fellow recruitment, and that a 2 year clinical track might have broader appeal to potential trainees. Nonetheless, the majority (65 %) of North American respondents thought the ideal training duration was 3 years, not two. Decisions to shorten training must be weighed against the importance of broad clinical exposure and the need to train clinician-scholars to advance the field. These data highlight the importance of tailoring training requirements to regional and country-level needs and expectations, without sacrificing clinical expertise [10].

### Increasing Job opportunities

Many respondents from Africa (46 %), Asia (27 %), Latin America (26 %), Australia/NZ (30 %), and the Middle East (23 %) reported that lack of resources was an obstacle to job acquisition. Lack of resources might signify inadequate, aging, or absent infrastructure, insufficient financial support on the part of institutions or governments, or inadequate number of allotted training slots or PN positions. For example, 30 % of Asian respondents reported that redundancy with adult nephrologists or general pediatricians was a concern. Similarly, lack of institutional or government support and resources was frequently mentioned by respondents from Africa (60 %), Asia (32 %), Latin America (33 %), and the Middle East (22 %) as an obstacle to trainee recruitment. Strengthening regional professional associations and their advocacy efforts should be made a high priority.

### Policy development, advocacy activities, and collaboration

Advocacy at government and ministry levels is also needed to support policy development and resource allocation that are favorable to both established and developing PN communities. We recommend utilizing World Kidney Day as a platform for such advocacy [21]. These interventions must be tailored to the political, social, and institutional environments and will vary by region and country. We also suggest that regional and international PN societies develop resources to assist with this form of advocacy. Interventions to bolster training opportunities and develop long term institution to institution support such as the ISN’s Sister Renal Center Program, IPNA’s fellowship training grants, and the annual International Pediatric Nephrology Fellows Conference are additional examples of ongoing efforts to support the PN workforce [11, 22–25].

### Other activities

Future assessments of the PN workforce might consider incorporating additional factors such as pediatric population size, rural versus urban disparities, PN training requirements, physician remuneration, and healthcare expenditure. Building a comprehensive and up-to-date database of practicing pediatric nephrologists, physician extenders, nurse practitioners, and dialysis personnel will be of integral to the success of these efforts. Other interventions to support the growth of PN workforce include developing and sharing novel teaching tools, strengthening existing trainee relationships, broadening and facilitating mentorship relationships across institutions, exposing potential trainees to nephrology early in their education, training nurse practitioner and mid-level providers, and engaging potential trainees in national level meetings and conferences. Journal editors and guideline committees should be committed to publish research, guidelines, and conference recommendations that are relevant and practical for PNs working in low resource settings. Initiatives to this effect, for example, include the recent KDIGO Implementation Strategies Conference on Understanding Needs in Low and Middle Income Countries [26].

To our knowledge, this is the first worldwide assessment of the PN workforce. Strengths of our study include its quantitative and qualitative assessments of the PN workforce and broad international reach. Furthermore, the geographic distribution of respondents closely mirrored that of non-respondents (see Table 1). Our results must be considered in the context of study limitations. Our response rate of 15 % is modest; however, this response rate is consistent with previous multinational web-based surveys of physicians [27–32]. Second, the survey did not differentiate between active (~1300) and inactive IPNA members, rendering it subject to participation by PNs no longer in active practice. Third, the survey was an English language, internet-based survey, resulting in possible under-coverage and response bias. The survey should be translated into other languages for future, broader evaluations of the PN global workforce. Fourth, selection bias, especially regarding free-text responses, is potentially operative in our data. We speculate the direction of bias to be towards greater workforce inadequacy. Finally, we recognize the high degree of variation likely to be found both across geographic regions and within individual countries. Future surveys should aim for higher response rates to allow for additional sub-regional analyses.

## Conclusions

These data provide a broad assessment of perceptions of the global PN workforce, and have potential implications for workforce planning, policy development, and advocacy campaigns. Efforts could include capitalizing on the elegance of our organ system by developing innovative and inspiring teaching tools, re-imagining ways of sharing responsibility and clinical care coverage to improve work-life balance, joining together to advocate for resources and recognition in developing nations, reigniting discussions regarding training duration and research requirements, and strengthening research and training partnerships across the globe. These goals should be accomplished while combating erosion of our scope of practice by other specialties and maintaining full dedication and service to our patients and their families.

## Abbreviations

IPNA, International Pediatric Nephrology Association; NZ, New Zealand PN, pediatric nephrology; PNs, pediatric nephrologists

## Additional files

Additional file 1: Survey Response Data. Description of data: Survey response data for perceived workforce adequacy, difficulty recruiting trainees, difficulty finding a job after training, optimal training duration, and mandatory research requirement. (PDF 438 kb) Additional file 2: CHERRIES Checklist. Description of data: Checklist for reporting results of Internet Surveys. (PDF 299 kb) Additional file 3: Global Pediatric nephrology Workforce Questionnaire. Description of data: Survey Instrument. (DOCX 16 kb)
