# Supplementary material for: The global pediatric nephrology workforce: a survey of the International Pediatric Nephrology Association
Source: BMC Nephrol. 2016 Jul 15;17:83. doi: 10.1186/s12882-016-0299-2 (PMC4946101; doi:10.1186/s12882-016-0299-2)
Supplement: Additional file 1: — Survey Response Data. Description of data: Survey response data for perceived workforce adequacy, difficulty recruiting trainees, difficulty finding a job after training, optimal training duration, and mandatory research requirement. (PDF 438 kb) [file 12882_2016_299_MOESM1_ESM.pdf]

Survey Response Data

Region	N	Optimal Years of Training: 1	2	3	4	>4
North America	107	0	35	69	2	1
Latin America	32	0	14	13	5	0
Europe	70	1	19	35	10	6
Asia	57	3	16	33	2	2
Africa	15	2	8	2	2	1
Australia / NZ	15	0	1	13	1	0
Middle East	25	0	5	18	1	1

Theme: Difficulty Obtaining A Job

Region	Lack of Resources	Low Salary	Lack of Job Positions	Unrecognized subspeciality or Adult Nephrologist / General Pediatrician Redundancy	Geogrpahic Location of Job Oppurtunities
Global Average n=213					
North America n=65	7	8	18	3	31
Latin America n=23	6	1	9	2	5
Europe n=46	9	3	32	2	9
Asia n=43	12	3	13	13	3
Africa n=13	6	0	5	1	1
Australia / NZ n=10	3	0	9	0	0
Middle East n=13	3	0	5	2	1

	Practice Type						
Region	Private Practice	Academia or University Based	Government Affiliated	Miliary Affiliated	Mulitple Practice Types	Other	Total
North Ame	6	94	1	0	2	4	107
Latin Amer	3	9	11	0	17	0	40
Europe n=7	0	23	33	0	16	0	72
Asia n=64	5	26	18	2	10	3	64
Africa n=1(	2	6	1	0	7	0	16
Australia /	1	1	10	0	1	2	15
Middle Eas	0	8	7	0	12	0	27

Percieved Workforce Adequacy

	Severe Shortage	Moderate Shortage	Mild Shortage	Adequate	Mild Surplus	Moderate Surplus	Severe Surplus	Total
North America	7	37	41	19	3	0	0	107
Latin America	8	7	8	9	0	0	0	32
Europe	3	11	16	32	7	1	1	71
Asia	16	21	6	11	2	0	0	56
Africa	11	3	1	0	0	0	0	15
Australia / NZ	0	2	1	4	5	2	1	15
Middle East	3	7	9	2	2	1	1	25

Themes: Difficulty Reruitnig Trainees

	Total	Low Job Availability	Lack of Institutional or Government Resources / Support	Lack of Trainee Interest	Lack of Training Positions	Low Salary	Hard Work / Poor Work-Life Bbalance
North America n=8[illegible]	88	5	5	41	2	36	31
Latin America n=21	21	1	7	4	3	6	2
Europe n=47	47	7	9	6	8	5	5
Asia n=47	47	3	15	9	4	7	11
Africa n=15	15	1	9	2	3	1	2
Australia / NZ n=10	10	3	0	2	4	1	0
Middle East n=18	18	1	4	2	3	1	4

Ease of Recruiting Trainees

	Very Difficult	Difficult	Somewhat Difficult	Neutral	Somewhat Easy	Easy	Very Easy	Total
North America	13	15	16	9	4	1	0	58
Latin America	1	3	6	6	3	1	0	20
Europe	3	7	9	7	8	8	4	46
Asia	2	5	16	5	2	3	0	33
Africa	2	2	1	0	0	3	1	9
Australia / New Zelan	0	1	2	3	4	3	0	13
Middle East	0	2	5	5	2	1	0	15

Difficulty Finding A Job After Training

	Very Difficult	Difficult	Somewhat Difficult	Neutral	Somewhat Easy	Easy	Very Easy	Total
North America	1	5	13	21	23	30	14	107
Latin America	3	7	10	3	3	5	1	32
Europe	5	10	18	21	11	6	0	71
Asia	4	9	15	10	3	9	6	56
Africa	0	3	0	2	3	5	2	15
Australia / New Zelan	1	7	6	1	0	0	0	15
Middle East	0	4	2	4	5	5	5	25

	Do you feel that research or scholarship shold be a mandatory componant of fellowship training	
	Yes	No
Global Average n=321	251	71
North America n=107	80	27
Latin America n=32	26	6
Europe n=71	46	25
Asia n=56	53	3
Africa n=15	14	1
Australia / NZ n=15	9	6
Middle East n=26	23	3

	Is research or scholarship a requierment for training in your country? N=301	
	Yes	No
Global Average n=321	190	111
North America n=107	102	4
Latin America n=32	15	14
Europe n=71	18	48
Asia n=56	32	21
Africa n=15	6	5
Australia / NZ n=15	7	6
Middle East n=25	10	13
