# Supplementary material for: The global pediatric nephrology workforce: a survey of the International Pediatric Nephrology Association
Source: BMC Nephrol. 2016 Jul 15;17:83. doi: 10.1186/s12882-016-0299-2 (PMC4946101; doi:10.1186/s12882-016-0299-2)
Supplement: Additional file 3: — Global Pediatric nephrology Workforce Questionnaire. Description of data: Survey Instrument. (DOCX 16 kb) [file 12882_2016_299_MOESM3_ESM.docx]

**Global Pediatric Nephrology Workforce Questionnaire**

**8/2015**

Dear Colleague,

This 15 question, 5 minute, survey will give us an opportunity to characterize training settings for pediatric nephrology around the world. The questions are for both trainees and fully trained pediatric nephrologists.  In appreciation for your time we will send a donation to the International Pediatric Nephrology Association.

Thank you

Maria Ferris, Mara Medeiros, William Primack, Dorey Glenn  and Adam Weinstein

In what country do you practice?

In what country did you complete your nephrology training?

What percentage of your time is devoted to clinical pediatric nephrology and research?

______ Clinical Pediatric Nephrology

______ Research

______ Other

Your pediatric nephrology program is best described as: (you may choose more than one)

- private practice
- Academia or in a university
- government affiliated hospital
- military affiliated hospital
- Other (please specify ____________________

Does your current program train pediatric nephrology fellows (sub-specialty residents or registrars)?

- Yes
- No

If yes, please answer how easy or difficult it is to:

|  | Very easy | Easy | Somewhat Easy | Neutral | Somewhat Difficult | Difficult | Very Difficult |
| --- | --- | --- | --- | --- | --- | --- | --- |
| Recruit pediatric nephrology trainees (fellows or registrars) |  |  |  |  |  |  |  |

What makes it easy or difficult to recruit nephrology trainees in your country?

In your country, how many years of training are currently required to become a pediatric nephrologist?

- One year
- Two years
- Three years
- Four years
- other

In you opinion, what is the optimal length for pediatric nephrology training?

- 1 year
- 2 years
- 3 years
- 4 years
- more than 4 years

Is there a certifying board in pediatric nephrology in your country?

- Yes
- No
- I do not know

What is your opinion of the adequacy of the pediatric nephrology workforce in your country?

- Severe shortage
- Moderate shortage
- Mild shortage
- Adequate
- Mild surplus
- Moderate surplus
- Severe surplus

After fellowship training, how easy or difficult  is it to find a job as a pediatric nephrologist in your country?

|  | Very easy | Easy | Somewhat easy | Neutral | Somewhat difficult | Difficult | Very difficult |
| --- | --- | --- | --- | --- | --- | --- | --- |
| Find a job as a pediatric nephrologist after training |  |  |  |  |  |  |  |

What are the challenges or obstacles to finding a nephrology position after training?

Is research or scholarship a requirement for nephrology training in your country?

- Yes
- No
- I am not sure

Do you feel that research or scholarship should be a mandatory component of nephrology training?

- Yes
- No

Please feel free to share any comments you may have regarding length of training below.
